# Nitrogen cycling during an Arctic bloom: from chemolithotrophy to nitrogen assimilation

**DOI:** 10.1128/mbio.00749-25

**Published:** 2025-05-12

**Authors:** Rafael Laso-Pérez, Juan Rivas-Santisteban, Nuria Fernandez-Gonzalez, Christopher J. Mundy, Javier Tamames, Carlos Pedrós-Alió

**Affiliations:** 1 Biogeochemistry and Microbial Ecology Department, Museo Nacional de Ciencias Naturales (MNCN-CSIC), Madrid, Spain; 2 Department of Systems Biology, Centro Nacional de Biotecnología (CNB-CSIC), Madrid, Spain; 3 Facultad de Ciencias Experimentales, Universidad Francisco de Vitoria, Pozuelo de Alarcón, Spain; 4 Centre for Earth Observation Science, Clayton H. Riddell Faculty of Environment, Earth, and Resources, University of Manitoba, Winnipeg, Canada; Georgia Institute of Technology, Atlanta, Georgia, USA

**Keywords:** nitrogen cycling, ammonia oxidation, phytoplankton bloom, arctic microbiology, nitrogen metabolism

## Abstract

In the Arctic, phytoplankton blooms are recurring phenomena occurring during the spring-summer seasons and influenced by the strong polar seasonality. Bloom dynamics are affected by nutrient availability, especially nitrogen, which is the main limiting nutrient in the Arctic. This study aimed to investigate the changes in an Arctic microbial community using omics approaches during a phytoplankton bloom focusing on the nitrogen cycle. Using metagenomic and metatranscriptomic samples from the Dease Strait (Canada) from March to July (2014), we reconstructed 176 metagenome-assembled genomes. Bacteria dominated the microbial community, although archaea reached up to 25% of metagenomic abundance in early spring, when *Nitrososphaeria* archaea actively expressed genes associated with ammonia oxidation to nitrite (*amt, amo, nirK*). The resulting nitrite was presumably further oxidized to nitrate by a *Nitrospinota* bacterium that highly expressed a nitrite oxidoreductase gene (*nxr*). Since May, the constant increase in chlorophyll *a* indicated the occurrence of a phytoplankton bloom, promoting the successive proliferation of different groups of chemoorganotrophic bacteria (*Bacteroidota, Alphaproteobacteria, Gammaproteobacteria*). These bacteria showed different strategies to obtain nitrogen, whether it be from organic or inorganic sources, according to the expression patterns of genes encoding transporters for nitrogen compounds. In contrast, during summer, the chemolithotrophic organisms thriving during winter reduced their relative abundance and the expression of their catabolic genes. Based on our functional analysis, we see a transition from a community where nitrogen-based chemolitotrophy plays a relevant role to a chemoorganotrophic community based on the carbohydrates released during the phytoplankton bloom, where different groups seem to specialize in different nitrogen sources.

**IMPORTANCE:**

The Arctic is one of the environments most affected by anthropogenic climate change. It is expected that the rise in temperature and change in ice cover will impact the marine microbial communities and the associated biogeochemical cycles. In this regard, nitrogen is the main nutrient limiting Arctic phytoplankton blooms. In this study, we combine genetic and expression data to study the nitrogen cycle at the community level over a time series covering from March to July. Our results indicate the importance of different taxa (from archaea to bacteria) and processes (from chemolithoautotrophy to incorporation of different nitrogen sources) in the cycling of nitrogen during this period. This study provides a baseline for future research that should include additional methodologies like biogeochemical analysis to fully understand the changes occurring on these communities due to global change.

## INTRODUCTION

The Arctic region is a unique environment characterized by extreme seasonal transitions between ice-covered winters and ice-free summers. The stress of these transitions has increased due to climate change affecting the whole ecosystem: from physicochemical variables to biological interactions. Scientists have observed an increase in phytoplankton biomass with subsequent higher net primary production (NPP) owing to the sea ice decline and a larger supply of nutrients into the Arctic system (1). Primary production is associated with recurrent phytoplankton blooms during the summer months, which are dependent on the availability of nutrients like nitrogen or phosphorus (2–4). Nitrogen is the primary nutrient affecting phytoplankton growth in the Arctic, since it becomes limiting after the spring-summer bloom (2, 3, 5–7). In recent years, the biogeochemistry of the Arctic nitrogen cycle and its relationship to primary productivity has been studied in more detail, showing that nitrogen derived from rivers and coastal erosion supports around 28%–51% of the Arctic NPP (8). Similarly, it has been reported that the majority of the NO_3_^-^ in Arctic waters is produced biologically *in situ* (9), and that nitrogen fixation by diazotrophs might be a more important process than previously thought in ice-free waters (10). Besides biogeochemistry, the emergence of sequencing technologies has opened new perspectives regarding the function of prokaryotes in the nitrogen cycle. By using 16S rRNA gene surveys, previous studies linked different taxonomic groups with specific nitrogen compounds (11). Omics technologies have gone beyond allowing the study of key genes and the corresponding organisms. For instance, a metagenomic analysis of the Arctic diazotrophic community has unraveled that nitrogen fixers are more abundant than previously thought and possess different variants of the *nifH* gene, the hallmark of the process (12). With a similar approach, Royo-Llonch et al. showed that organisms with the potential for nitrification, ammonia-oxidizing archaea (AOA) and bacteria, and nitrite-oxidizing bacteria (NOB) are more prevalent in spring and autumn, when chemolithotrophic prokaryotes increase in importance (13). Similarly, some Arctic clades of AOA have been suggested to use urea to fuel nitrification (14), although the contribution of urea to the local nitrogen pool is still unclear (15, 16). Nitrate can also serve as a nitrogen source in the Arctic, whereas the presence of nitrate dissimilatory genes in the oxic Arctic environments has been interpreted as “strategy of metabolic versatility” (17). Other organic nitrogen compounds like taurine, polyamines, or amino acids might also serve as nitrogen (and energy) sources (18, 19), but little is known about the corresponding transporters and mechanisms from an omics perspective in the Arctic. Very few omics studies have investigated nitrogen cycling from a community perspective, especially in some areas like the Canadian Arctic Archipelago, making it difficult to understand the local microbial communities and how they can be affected in the current scenario of climate change (20). In this regard, a metagenomics study from this area captured spatial differences, although not significant, between seawater and sea ice in relation to different functions like nitrate and nitrite cycling (21). Most omics studies in the Arctic lack expression and time series data, impeding a better understanding of the transcriptional response of microbial communities to the seasonal changes. Here, we wanted to characterize the nitrogen cycle in a prokaryotic Arctic community during the seasonal winter-summer transition using a unique combination of genomic and expression data over a period of 5 months (March–July 2014). Previous studies on this data set have analyzed how bacterioplankton competed for iron during this period (22), and how phytoplankton proliferation triggered the growth of *Bacteroidota* with the ability to degrade carbohydrates (23). However, these two studies did not reconstruct genomes to resolve species- and strain-level functional traits. Using the same data set, we have now reconstructed metagenome-assembled genomes (MAGs), which allowed us to link taxonomic identity with several metabolic traits. MAG reconstruction and expression data have already been used to study microbial communities in the Arctic (13). However, the data set of our study is unique since it includes expression information of the same location (Dease Strait, Canadian Arctic) for a 5-month period, which, combined with the MAG-based approach, provides a higher-resolution view of how individual microbial species contribute to nitrogen cycling. By integrating genomic information with expression data, we uncovered the ecological succession from nitrification to a summer bloom situation, when clade-specific transcriptional strategies are expressed for nitrogen utilization.

## RESULTS

### Community composition

**Fig 1:**
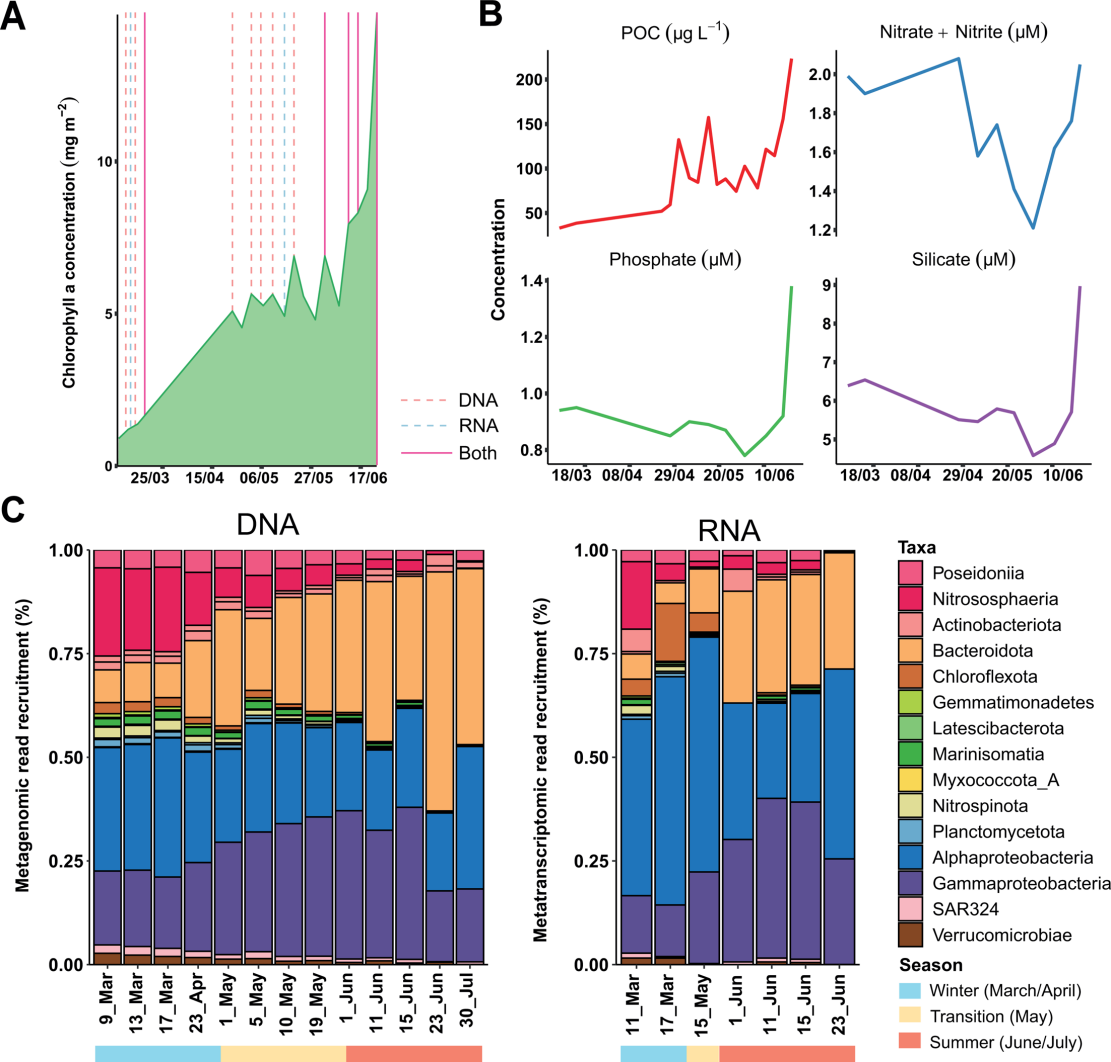
Overview of the environmental conditions during the sampling and the recruitment of DNA and RNA reads by the MAGs. (A) Graph showing the chlorophyll *a* concentration over time (day/month). The increase in chlorophyll indicates a phytoplankton bloom. Vertical lines indicate when DNA and RNA libraries were collected. (B) Particulate organic carbon (POC) and nutrient concentrations over time. (C) Normalized fraction of the DNA and RNA reads recruited per MAG grouped by taxa. The fraction of DNA or RNA reads that was not recruited from the MAGs is shown in Table S2, and it was approximately 50%.

Using 13 metagenomic samples retrieved from the Dease Strait (Canadian Arctic; Fig. S1) from March until July 2014 (Table S1), we reconstructed 176 MAGs (Table S2). During the sampling period, a phytoplankton bloom developed as shown by the increase in chlorophyll *a* concentration and particulate organic carbon (Fig. 1A; Tables S3 and S4). Nutrients decreased in concentration during the bloom, especially nitrate and nitrite, and then increased at the end of June (Fig. 1B; Table S4).

Read recruitment analysis showed that our MAGs captured approximately half of the metagenomic reads across all samples (Table S2, row 5). According to the genomic relative abundance (Fig. 1C; Table S2), the dominant groups were ammonia-oxidizing archaea from the class *Nitrososphaeria* and the bacterial groups *Alphaproteobacteria, Gammaproteobacteria,* and *Flavobacteriaceae* (*Bacteroidota*). Community composition was similar at the RNA level (Fig. 1C) and based on the mapping of metagenomic 16S rRNA gene reads using phyloFlash (Fig. S2; see Materials and Methods). Bacteria dominated during the whole period, although archaea reached up to 25% of metagenomic coverage in March, when a *Nitrososphaerales* MAG (Nitrosopumilus_01; completeness [comp.] 92.6%, contamination [cont.] 0%) was the most abundant organism of the whole data set (19%–21%) and decreased in abundance during the spring. In contrast, archaeal MAGs from the order *Poseidionales* showed lower relative abundances, although some seasonal changes were observed with higher relative abundances during winter-spring (4%–6% vs 2%–3% in summer). Several bacterial groups had a strong seasonality presumably linked to the phytoplankton bloom. For instance, *Flavobacteriales* MAGs (class *Bacteroidia*) represented 7% of the prokaryotic assemblage in March and reached 57% in late July, at the peak of the bloom. Other seasonal groups were the alphaproteobacterial *Pelagibacterales* and the gammaproteobacterial *Pseudomonadales*, PS1 (that includes the family *Thioglobaceae*), and SAR86. The gammaproteobacterial groups increased their abundance during May to peak in June and decreased during the last period of the sampling. In contrast, the alphaproteobacterial *Pelagibacterales* slowly decreased in their relative abundance during middle spring to increase dramatically at the end of July (27.5%). These changes were mostly associated with a single MAG (Alphaproteobacteria_10; comp. 63.8%, cont. 0%), which always showed a high relative abundance (>6%).

Most RNA reads mapped to a few MAGs in all the samples (Table S2), like a *Pelagibacterales* MAG (Alphaproteobacteria_10) that captured more than half of the mapped RNA reads during mid-March and May. During winter, the archaeal *Nitrosopumilus* MAG captured 16% of the transcripts. In contrast, two gammaproteobacterial MAGs, a PS1 (Gammaproteobacteria_11; comp. 90.3%, cont. 0.1%) and a *Pseudomonadales* (Gammaproteobacteria_22; comp. 100%, cont. 0%), increased their transcriptomic recruitment from 5% and less than 1%, respectively, in winter to more than 10% in May–June. In *Bacteroidota*, no MAG captured over 10% of the transcripts, but still two *Flavobacteriales* recruited 5%–6% of the RNA reads in May–June.

**Fig 2:**
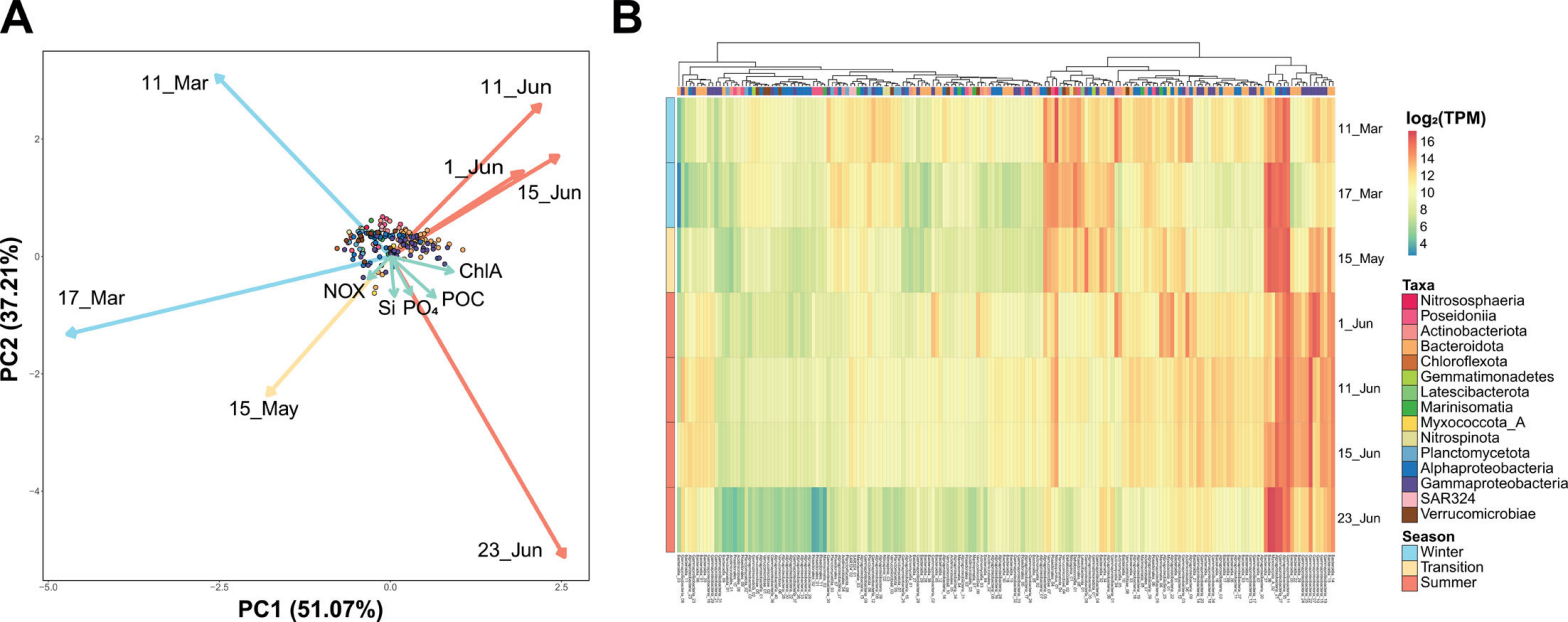
Variability in prokaryotic community transcription based on MAGs across sampling dates. (A) RDA constrained by environmental variables. Each dot represents a MAG. Arrows indicate samples (blue for winter, yellow for transition, and red for summer) and environmental variables (green-blue). (B) Hierarchical clustering and heatmap of the log-normalized expression of each MAG (columns). Rows indicate sample date. For both panels, color indicates taxonomy at the class or phylum level.

A redundancy analysis (RDA) showed that the expression profiles of *Gammaproteobacteria* and *Bacteroidota* MAGs seemed to be more associated with June samples, while *Nitrososphaeria* and *Nitrospinota* transcription patterns were associated with March time points (Fig. 2A). In fact, the transcriptomic profile of the 176 MAGs showed changes in expression from winter to summer, with different groups of MAGs dominating in early March, in the May transition, in early and mid-June peak, and at the end of June (Fig. 2B). Interestingly, the late June profile differed from the rest of June samples, showing a different expression pattern that occurred when nutrient concentrations recovered (Fig. 1B).

### Functional analysis

**Fig 3:**
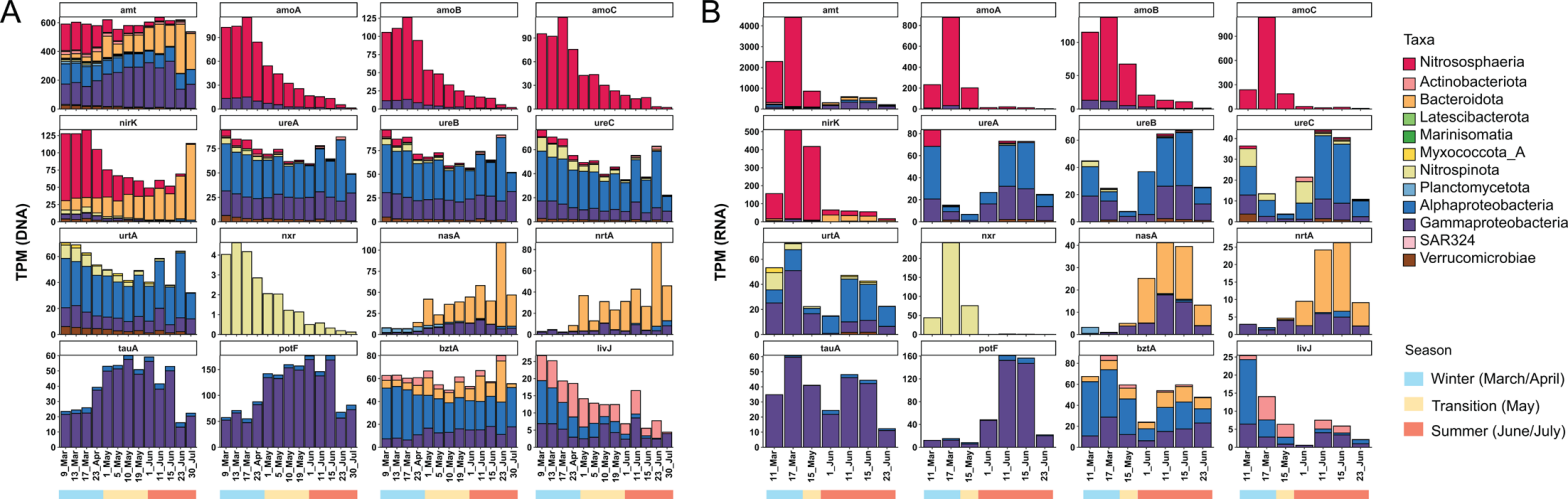
Transcripts per million (TPM) values of the nitrogen-cycling genes. (A) TPM in the DNA libraries of the different genes grouped by taxonomical class or phylum. (B) TPM in the RNA libraries of the different genes grouped by taxonomical class or phylum.

To study the nitrogen cycling within our data set, we searched and classified key genes of the different nitrogen-cycling processes in each MAG (see Table 1 and Materials and Methods). Our analysis showed a transition from a community highly expressing mechanisms for nitrification to another where different nitrogen assimilation mechanisms were transcribed (Fig. 3; Table S5).

**TABLE 1:** Overview of the nitrogen-cycling genes studied in this article

Gene	Enzyme	Process
*nifH*	Nitrogenase subunit H	Nitrogen fixation
*amt*	Ammonium transporter	Ammonia transport into the cell
*amoABC*	Ammonia monooxygenase	Ammonia oxidation (nitrification)
*nirK*	Dissimilatory nitrite reductase (NO-forming)	Denitrification (ammonia oxidation in AOA)
*nirS*	Dissimilatory nitrite reductase (NO-forming)	Denitrification
*ureABC*	Urease subunit alpha	Urea degradation (hydrolysis pathway)
*uca*	Urea carboxylase	Urea degradation (carboxylating pathway)
*narG*	Nitrate reductase subunit G	Dissimilatory nitrate reduction (anaerobic)
*nxrA*	Nitrite oxidoreductase subunit A	Nitrite oxidation (nitrification)
*nasA*	Assimilatory nitrate reductase	Assimilatory nitrate reduction
*nosZ*	Nitrous oxide reductase	Denitrification
*hzsA*	Hydrazine synthase subunit A	Anammox (anaerobic oxidation of ammonia)
*nrtA*	Nitrate/nitrite transport system substrate-binding protein	Nitrate transport into the cell
*tauA*	Taurine transport system substrate-binding protein	Taurine transport into the cell
*potF*	Putrescine transport system substrate-binding protein	Putrescine transport into the cell
*bztA*	Glutamate amino acid transport system substrate-binding protein	Amino acid transport into the cell
*livJ*	Branched-chain amino acid transport system substrate-binding protein	Amino acid transport into the cell

### Nitrification-related genes are highly expressed during winter-mid-spring

Transcriptomic analysis revealed high expression levels for genes related to nitrification (*amoABC* and *nxr*) from March until May. Two MAGs in our data set, a *Nitrososphaeria* (Nitrosopumilus_01) and a *Burkholderiales* (Gammaproteobacteria_04; comp. 96.2%, cont. 0.2%), encoded a complete Amo enzyme, the hallmark enzyme of ammonia oxidation. Only the Nitrosopumilus_01 MAG showed high genomic relative abundance, as well as high gene expression. Specifically, during winter, the *Nitrososphaeria* had extremely high transcriptomic levels, with an expression peak for the three *amo* genes in mid-March (although *amoB* showed lower expression levels), followed by a decrease and almost no expression signal in June (Fig. 3B). Other *Nitrososphaeria* genes involved in ammonia oxidation showed a similar transcription pattern to the gene encoding NirK, as well as the transmembrane ammonia transporter gene *amt* (Fig. 3). Interestingly, genes encoding a urease enzyme (Ure) and a subunit of the urea transporter (UrtA) were also present in the Nitrosopumilus_01 MAG, but showed low transcription levels across all RNA samples.

**Fig 4:**
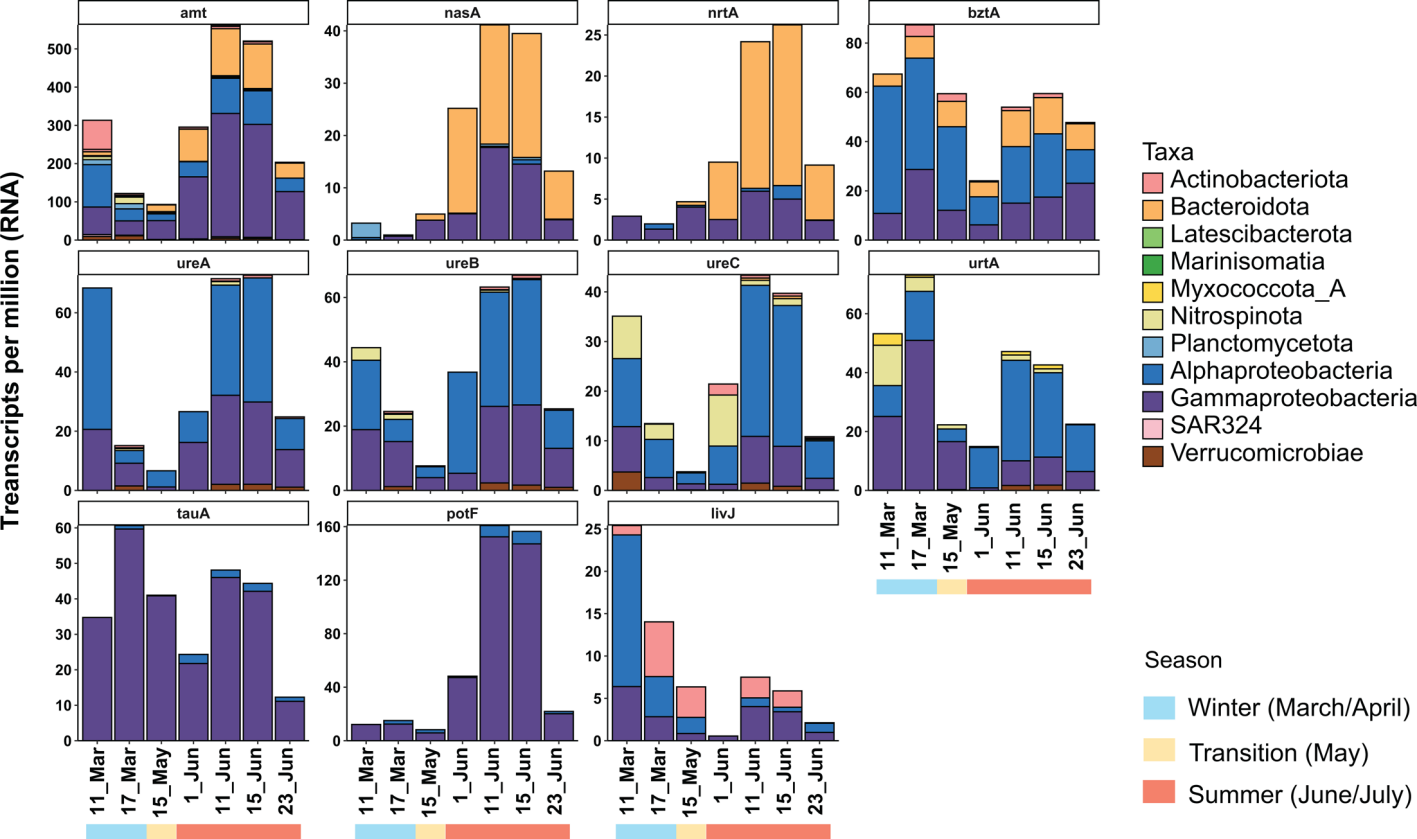
Bacterial expression of the studied nitrogen-cycling genes based on transcripts per million values. Archaeal genes have been removed from the plot for clarity. The color indicates taxonomy at the class or phylum level.

The *nxrA* gene, encoding a subunit of the Nxr enzyme, is only encoded by one MAG of the bacterial class *Nitrospinia* (Nitrospinia_01; comp. 89.8%, cont. 3.4%). Although this MAG did not recruit more than 1% of the RNA reads, its *nxrA* gene showed intense transcription during winter in a similar fashion to the genes of the ammonia-oxidizing archaea (Fig. 3). There were two additional *Nitrospinia* MAGs affiliated to a different genus (Nitrospinia_02; comp. 59.6%, cont. 3.6%; and Nitrospinia_03; comp. 53.2%, cont. 2.9%)*,* in which we could not detect genes for the Nxr complex. Instead, both genomes encoded for a Ure, absent in the Nitrospinia_01 MAG (Table S2). The *ureC* gene of Nitrospinia_03 showed moderate expression levels in March and early June (Fig. 4; Table S5).

### Bacteria thriving in summer express diverse mechanisms to use organic and inorganic nitrogen

With the winter-summer transition, there was a shift in the prokaryotic community with a substantial increase in heterotrophic bacteria feeding on the carbohydrates released by the phytoplankton (23). The corresponding MAGs possess genes encoding different transporters and enzymes for the utilization of diverse nitrogen compounds. The taxonomy and expression profiles of these genes changed during the studied period, suggesting a succession of strategies for nitrogen assimilation related to the proliferation of specific bacterial taxa (Fig. 4).

### Expression of inorganic nitrogen assimilation genes

**Fig 5:**
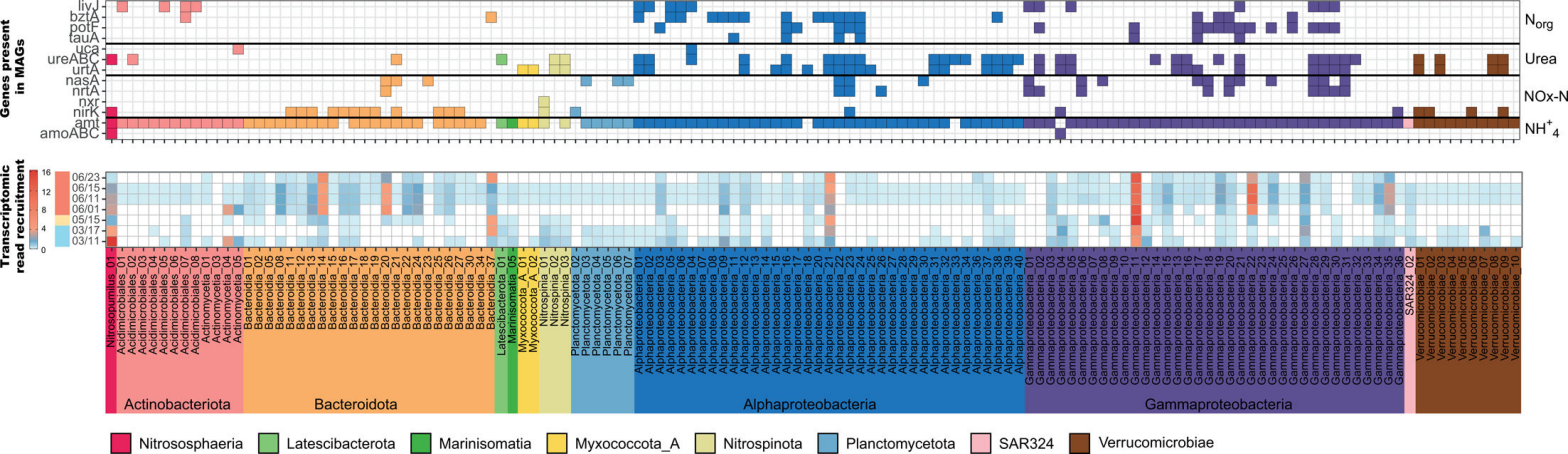
Overview of the nitrogen-related genes present in the MAGs. Each column of both panels represents a MAG. Only MAGs with at least one of the genes from Table 1 are shown in the figure. Top panel tiles indicate the presence of the corresponding gene in the MAG. The color in the top panel refers to the class or phylum, and rows indicate the name of the gene(s) which are grouped by metabolisms: organic nitrogen, urea, nitrate/nitrite or ammonia. Bottom panel indicates the fraction of RNA reads mapping to the corresponding MAG. Each row shows a different date (as indicated on the left).

Most MAGs in our data set had genes for an ammonium transporter (Amt, Fig. 5). During winter, the archaeal *amt* genes from *Nitrososphaeria* represented 90% of all *amt* transcripts (Fig. 3B), but in June, archaeal *amt* expression plummeted, while bacterial *amt* genes were highly transcribed, especially those of the most abundant groups: *Gammaproteobacteria, Alphaproteobacteria,* and *Bacteroidota* (Fig. 4). In fact, the RNA:DNA ratio for *amt* genes showed a constant increase during the summer months that exceeded the values of March (Fig. S3).

On the contrary, we did not find any canonical *nifH* gene in all our MAG data sets. There was a *nifH-*like gene in the Alphaproteobacteria_22 MAG (comp. 92.2%, cont. 2.8%) affiliated to the group V (former group IV), which includes genes associated with photosynthesis pigment biosynthesis and are not involved in nitrogen fixation (24, 25). The contiguous genes of this *nifH*-like gene were also related to pigment biosynthesis (Table S6). We searched for *nifH* genes in the non-binned fraction of the assembly to discard the possibility that *nifH* genes were not included in the bins. We found nine *nifH-*like genes, which were again affiliated to group V (Table S6).

Genes encoding the Nrt system, the ABC transporter for nitrate/nitrite, were present in 13 MAGs affiliated to the *Alphaproteobacteria, Gammaproteobacteria,* and *Bacteroidia* (Fig. 5). Nevertheless, expression of the corresponding *nrtA* gene is almost circumscribed to a single MAG during summer (Fig. 4), specifically a *Bacteroidia* organism (Bacteroidia_20; comp. 84.4%, cont. 0%), which according to genome coverage thrives in June, reaching 4%–5% relative abundance (Table S2). In fact, this *Bacteroidia* MAG also highly expresses a *nasA* gene in June (Fig. 4), which encodes the catalytic subunit of the assimilatory nitrate reductase. Nineteen additional genomes from the *Bacteroidia, Gammaproteobacteria, Alphaproteobacteria,* and *Plantomycetota* encoded for an assimilatory nitrate reductase (Nas), in most cases co-occurring with *nrtA* genes in the genome, pointing to a canonical assimilatory function (Fig. 5). These *nasA* genes showed again a differential transcription pattern with high expression in June dominated by two organisms: a gammaproteobacterium (Gammaproteobacteria_24; comp. 97.4, cont. 0.1%) and a *Bacteroidia* genome with three *nasA* variants (Bacteroidia_21; comp. 74.2%, cont. 3.2%).

### Expression of organic nitrogen utilization genes

**Fig 6:**
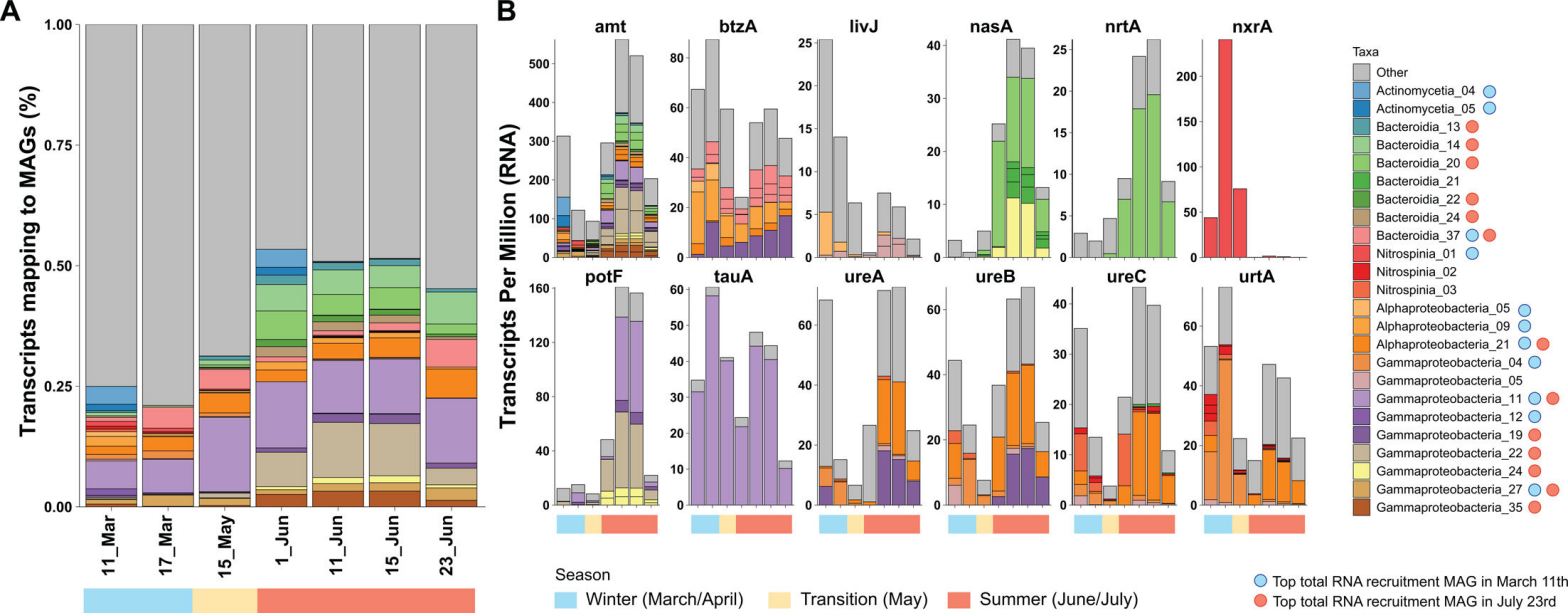
Expression profiles of a selection of bacterial MAGs. Each color indicates a MAG, and the gray section indicates the grouped values for the rest of the MAGs. A blue or a red circle next to the MAG name indicates that this organism is at the top of total RNA read recruitment of the whole MAG data set in winter (11/03) or summer (23/07). (A) Fraction of RNA transcripts mapping to the MAGs by date. (B) Expression profiles for a selection of nitrogen-cycling genes based on transcripts per million. Archaeal variants are excluded.

Around 22% of the MAGs of our data set encoded an enzyme for the degradation of urea. Most of the MAGs (34) coded for the Ure system, while only two MAGs harbored a *uca* gene, which showed almost no expression. The *ure* genes showed high levels of expression at two points: March and June. A similar expression pattern was shown by the *urtA* genes, which code for the substrate-binding protein of the urea transport system. Up to 35 MAGs possessed at least a copy of *urtA*. Both *ureABC* and *urtA* genes showed similar taxonomic expression profiles with a dominance of genes from *Alphaproteobacteria*, and to a lesser extent from *Gammaproteobacteria*. In mid-June, the Alphaproteobacteria_21 (comp. 98.3%, cont. 0.5%) MAG dominated the *ureABC* metatranscriptomic profiles, while in March, the Nitrospinia_03 MAG showed considerable levels of expression for the *ureBC* and the *urtA* genes (Fig. 6). In general, the DNA:RNA ratio of urea genes increased during June, falling at the end of the month.

Regarding other organic nitrogen compounds, we studied the presence and expression of genes encoding the substrate-binding protein of different ABC membrane transporters: *tauA* for taurine, *potF* for polyamines (like putrescine and spermidine), and *livJ* and *bztA* for different amino acids. Around 10% of the MAGs coded for amino acid or putrescine transporters, while only seven MAGs (4%) encoded the *tauA* gene. These mechanisms were mostly present in MAGs affiliated to *Gammaproteobacteria* or *Alphaproteobacteria*, except for some *Actinobacteriota* and *Bacteroidota* with *bztA* and *livJ* genes (Fig. 5; Table S2). According to transcriptomic data, only a few of these MAGs expressed these mechanisms. For instance, a *Thioglobaceae* MAG (Gammaproteobacteria_11) monopolized the expression of the *tauA* gene during the whole studied period with peaks in March and mid-June (Fig. 6). This MAG also dominated the expression profile of *potF* in combination with a *Pseudomonadales* (Gammaproteobacteria_22). In the case of *potF*, expression was almost restricted to June (Fig. 6). The transcriptomic profile of *bztA* was similar to that of *tauA* with expression peaks in March and June. Nevertheless, the *bztA* profile presented more taxonomic diversity, although a considerable fraction of the transcripts were affiliated to a *Pelagibacterales* MAG (Alphaproteobacteria_09; comp. 90.6%, cont. 0.5%), a gammaproteobacterium (Gammaproteobacteria_19; comp. 89.7%, cont. 0%), and a flavobacterium (Bacteroidia_37; comp. 59.6%, cont. 3%), the last one encoding three *bztA* genes. Opposite to the previous genes, the community profile of *livJ* showed a small expression in March and then decreased. Like *bztA*, this transcription profile was more diverse and not dominated by a single organism (Fig. 3).

### Anaerobic nitrogen-cycling genes

We detected a few genes related to anaerobic nitrogen processes, but they showed almost no expression (Fig. S4). For instance, a *Pseudomonadales* MAG (Gammaproteobacteria_28; comp. 97.6%, cont. 0.3%) harbors genes for a denitrification pathway (*narG, nirS, nosZ*). This MAG also encodes different transporters of nitrogen compounds (Amt, NrtA, LivJ, PotF, UrtA) or the urease enzyme, but none of these genes showed high levels of expression. An additional *Pseudomonadales* MAG (Gammaproteobacteria_21; comp. 97.8%, cont. 2.6%) had a different *nosZ* gene, but this gene did not recruit any transcript. Similarly, we only detected an *hzsA* gene, which encodes a hydrazine synthase, the marker for the anaerobic oxidation of ammonia. The gene belonged to a *Planctomycetota* MAG (Planctomycetota_03; comp. 94.2%, cont. 1.1%) and had almost no expression (Fig. S4).

### Autotrophy

We analyzed the transcription of different marker genes for carbon fixation (see Materials and Methods, Tables S7 and S8; Fig. S5). We detected the genes for rubisco (*rbcL* and *rbcS*) and phosphoribulokinase (*prk*) of the Calvin–Benson–Bassham cycle (CBB), for the ATP-dependent citrate lyase (*acl*) from the reverse tricarboxylic acid cycle, for the mesaconyl-CoA isomerase (*mct*) of 3-hydroxypropionate (3HP) bi-cycle, and for the 4-hydroxybutyrate dehydrogenase (*hbd*) from the 4-hydroxybutyrate/3HP cycle. We could not detect a carbon monoxide dehydrogenase/acetyl-CoA synthase, the key enzyme of the Wood–Ljungdahl pathway. The detected genes were present in 37 MAGs, with 20 of them containing genes for the Mct enzyme. These *mct* genes were expressed during the summer months, and their taxonomic profile was diverse, with a majority of variants affiliated to *Bacteroidota* and *Gammaproteobacteria*. In contrast, the expression of other autotrophic genes was dominated by specific taxa. The CBB genes affiliated to *Gamnmaproteobacteria* and were highly expressed in June. The *acl* and the *hbd* genes were both highly expressed in early March and corresponded respectively to *Nitrospinota* and *Nitrososphaeria*.

## DISCUSSION

We have reconstructed 176 MAGs of an Arctic microbial community from the Canadian Archipelago and studied their expression over 4 months with a special focus on nitrogen-cycling genes. The predominant clades (*Nitrososphaeria, Alphaproteobacteria, Gammaproteobacteria,* and *Bacteroidota*) were similar to those detected in previous 16S rRNA gene surveys in the Arctic Archipelago (21, 26–31). With the winter-summer transition, there is a shift in the prokaryotic community with the growth and decay of specific groups associated with the seasonal changes (i.e., melting of sea ice, increase in solar irradiance) and the phytoplankton bloom that developed since May (Fig. 1), as has been previously described (23). The majority of the spring-summer microorganisms are heterotrophic bacteria feeding on the carbohydrates released by the phytoplankton (23). These changes reflect on the community composition at the DNA and RNA level (Fig. 1) and the predominance of specific MAGs (Fig. 2). Thus, the association of the expression profiles from *Gammaproteobacteria* and *Bacteroidota* MAGs with June bloom samples is likely attributed to their ability to utilize the carbohydrates released by the phytoplankton (23). The June samples showed lower concentrations of different nutrients, including nitrate and nitrite (Fig. 1B; Table S4), which might be linked to its consumption, owing to phytoplankton growth. Interestingly, the community expression profile at the end of June seemed to be different from the rest of the June samples (Fig. 2B) and might be linked to a collapse of the microbial community associated with the bloom since concentrations of different nutrients increased at that point. Regarding the nitrogen cycle, the winter community had higher relative abundances of chemolithotrophic organisms expressing nitrification genes like *Nitrososphaeria* and *Nitrospinota*, while summer bacteria seemed to have an increased expression of different mechanisms for nitrogen assimilation. These results are limited by our sequence-based methodologies and would need additional future studies including biogeochemical measurements.

### High expression of genes from *Nitrosopumilus* and *Nitrospinia* suggests intense nitrification in winter

Genes involved in nitrification from Nitrosopumilus_01 and Nitrospinia_01 MAGs showed high expression from March until May, when their transcription plummeted. Although transcription does not always imply that a process is occurring, laboratory experiments have shown correlation between the expression of these genes in AOA and NOB cultures and the different steps of nitrification: ammonia and nitrite oxidation, respectively. Specifically, different experiments with AOA cultures have reported high transcription of *amoAB, nirK,* and *amt* genes in the presence of ammonium, while their expression decreased under ammonium limitation (32–34). On the contrary, *amoC* seems to always keep high levels of transcription, which has been linked to recovery from ammonia starvation (33, 35). Similarly, NOB cultures of *Nitrobacter* and *Nitrospira* had high transcription levels of *nxr* genes (36, 37), and the *nxrB* gene of *Nitrospira* seemed to be downregulated under nitrite starvation (38). Although our data set only contains NOB affiliated to *Nitrospinia*, a taxonomically different nitrite-oxidizing group, transcription regulation mechanisms might be similar in different NOB. Therefore, the high expression of *amoABC, nirK, amt,* and *nxr* genes in our samples strongly indicates that nitrification seems to be a prevalent metabolism occurring during winter, while it disappears in summer.

Many environmental studies have shown a correlation between archaeal *amoA* gene abundances and nitrification rates in the ocean (39–41). A study of Arctic waters reported the highest *amoA* gene abundances in winter, when potential nitrification rates and ammonium concentrations were also highest (42). This study suggested that ammonia oxidation in the Arctic prevails during winter due to higher concentrations of ammonia (3, 42), lack of competition with phytoplankton, and darkness that avoids photoinhibition (42, 43). In fact, AOA are prevalent in Arctic surface waters during winter and then disappear in summer (14, 44, 45). In our study, AOA and the *amoA* gene abundance were also highest in winter before the bloom (Fig. 3A), when there was no light. Likewise, a study in Antarctic surface waters measured higher nitrification rates correlated to higher archaeal *amoA* gene abundances during winter, although correlation was not significant for *amoA* transcripts. Authors hypothesize that this might be caused by rapid RNA degradation (46). Interestingly, in the same study, *Nitrospina* abundances covaried with *Nitrososphaeria* and correlated with ammonia oxidation (46), suggesting a metabolic coupling between both organisms in which AOA oxidize ammonia and produce nitrite, which is then oxidized by NOB. This co-occurrence also occurs in other marine systems including Arctic waters (44, 47–50). Both AOA and NOB are usually described as chemolithoautotrophs. A metaproteomic assessment of surface Antarctic waters only detected proteins for nitrification and carbon fixation of *Nitrososphaeria* and the NOB group *Nitrospira* in the winter season, pointing out that nitrification-based chemolithoautotrophy presents a distinct seasonal pattern in polar waters (51). We observed a similar trend in our data set with high abundances and high expression of autotrophic genes affiliated to *Nitrospina* and *Nitrososphaeria* and during winter, such as the reverse tricarboxylic acid cycle and 3-hydroxypropionate/4-hydroxybutyrate cycle, respectively (Fig. S5).

### Urea-based nitrification did not seem to be prevalent in this system

Physiological studies have shown the ability of some clades of marine AOA to use urea (52, 53), although this metabolism is not present in all strains of *Candidatus Nitrosopumilus* (54). Some of these studies have shown a poor correlation between urea-based nitrification and *ureC* expression (32, 40, 52), while a recent article indicated that urea triggered higher expression on urea-utilizing genes in AOA cultures in the absence of ammonia (53). Therefore, it is unclear if the expression of *ure* genes can serve as a proxy of urea-based nitrification by AOA. A previous study claimed that polar members of the AOA usually possess ureases, suggesting that urea might be fueling nitrification in Arctic waters when ammonia is low or intermittent (14). Nevertheless, additional environmental studies have shown that urea seems not to play a major role in nitrification in Arctic waters (16, 40), while its contribution in the Antarctic Ocean might be considerable, but less than ammonia, as suggested by physiological and stoichiometric measurements (40, 46, 55). The lack of biogeochemical data hampers our understanding of the role of *ure* genes present in the Nitrosopumilus_01 MAG, but based on the previous research on the Arctic and the low expression of *ure* genes, we hypothesize that urea was likely not playing a major role in nitrification in our system. Future biogeochemical studies coupled to omics approaches are needed to understand the role of urea in nitrification in the Arctic and how this reflects at the molecular level.

### Ammonia and nitrate seem to be the prevalent inorganic nitrogen sources

With the spring-summer transition and the corresponding phytoplankton bloom, the expression of nitrification genes decreased, while the transcription of different bacterial machineries for nitrogen utilization increased (Fig. 3). Ammonia seemed to be one of the preferred nitrogen sources, since most MAGs had an *amt* gene, and these genes were the most transcribed in June from all the studied genes, showing high RNA:DNA ratios (Fig. S3). Ammonia concentrations are usually low in the Arctic (3, 40, 42), but high expression of *amt* genes has been found in different marine oligotrophic environments (56–60). Therefore, this high expression pattern is proposed as an ecological strategy to scavenge low concentrations of ammonium (61, 62). For instance, peaks in ammonia concentration have been reported in earlier studies on Arctic phytoplankton blooms followed by a rapid turnover (63), indicating that ammonia can play an important role as a nitrogen source during Arctic bloom (64).

Genes for other nitrogen mechanisms were actively expressed in June. Up to 10% of the MAGs possessed *nrtA* or *nasA* genes for nitrate incorporation, although the Bacteroidia_20 MAG monopolized their expression profile. Nitrate is considered a key nitrogen source in the early stages of phytoplankton blooms in Arctic surface waters (65–67) until it gets exhausted in summer due to high rates of microbial uptake (68–70), which can account for a large fraction (25%–40%) of the total NO_3_^-^ uptake (2). Surprisingly, the Bacteroidia_20 seemed to deploy additional strategies to incorporate nitrogen since it was also expressing an *amt* gene, which could suggest that different nitrogen inventories were used to fulfil the corresponding nutrient needs.

The role of diazotrophy in the Arctic Ocean remains unclear (2), and calculations estimated that 1%–17% of new primary production relies on nitrogen fixation (71). In fact, nitrogen fixation has been measured in different Arctic regions (2), including the Canadian Arctic where rates reached up to 0.46 nmol N L^−1^ d^−1^ in open waters (72). Besides, molecular surveys have reported a considerable diversity of Arctic *nifH* genes (72–76). In our study, we could not identify canonical *nifH* genes, while we found *nifH-*like genes associated with the synthesis of bacteriochlorophylls in one MAG and in the unbinned fraction. Similarly, previous metagenomics analyses have not detected *nifH* genes in certain Arctic regions (13, 77). The absence of *nifH* genes in our data set might also be attributed to low-sequencing coverage, or due to seasonal distributions of diazotrophs, as has been suggested for cyanobacterial nitrogen fixers in the Central Arctic (73). Recent investigations have pointed out that some diazotrophs live in close symbiosis with algae (78) or even the existence of a nitrogen-fixing organelle or “nitroplast” in an algal cell (79). Since our libraries excluded eukaryotic organisms, we cannot rule out that some diazotrophs were discarded due to their close association with eukaryotes. Finally, a previous study on this data set proposed a potential competition for iron among the bacterioplankton (22). The hypothetical iron limitation of these waters could also explain the absence of diazotrophs.

### Many bacteria encoded and expressed different mechanisms to incorporate organic nitrogen

One-third of our MAGs possessed genes involved in the cycling of organic nitrogen. Most of these genes presented a similar community expression profile with a “V” shape: high expression in March, a sharp decrease in the May–June transition, and a transcription peak in mid-June. The May–June decrease might reflect a shift from the winter to the summer microbial community associated with the phytoplankton bloom. Thus, in June, different microorganisms would modulate transcription according to the summer situation and changes in the availability of different substrates.

Urea can serve as a nitrogen, carbon, and energy source. In our data set, the transcription peak of *ureABC* in mid-June could suggest an intense usage of urea by *Alphaproteobacteria* and *Gammaproteobacteria* during the bloom. Arctic urea concentrations are usually below 1 µM, but tend to be higher in summer (16, 80–82), probably due to the release of the urea accumulated in the winter ice (70). In line with this fact, Royo-Llonch reported transcription of urease genes in summer samples for several Arctic MAGs (13), and summer urea uptake has been measured in different Arctic waters (16, 80, 82). However, an isotope labeling experiment in Alaska waters demonstrated that in summer, bacteria and archaea tend to incorporate ammonia, while in winter, the usage of urea increased (82). In this study, urea-utilizing bacteria were affiliated to *Burkholderales* (old *Betaproteobacteria*), *Pelagibacterales* (*Alphaproteobacteria*), and *Firmicutes*. Indeed, urea genes were also highly expressed in our winter samples, including some affiliated to two *Burkholderales* (MAGs Gammaproteobacteria_04 and Gammaproteobacteria_05; comp. 98.9%, cont. 0%), which recruited up to 1% of the RNA reads during winter (Table S2). Besides, the Nitrospinota_03 MAG, which reached up to 0.7% metagenomic abundance in winter, also expressed urea-cycling genes in March. Therefore, we hypothesize that urea might serve as an energy and nutrient source for some winter organisms, while in summer, transcription of urease genes remains high to scavenge low concentrations of urea in the context of nitrogen limitation associated with the bloom.

Marine eukaryotes naturally produce taurine and polyamines, which can serve as a nitrogen source for prokaryotes when they are released (83–86). Biogeochemical and molecular studies have pointed to heterotrophic alphaproteobacteria from the *Pelagibacterales* (previously known as SAR11 group) and *Roseobacterales* as the main microorganisms using these substrates in the ocean (18, 51, 83, 84, 87–91). Strikingly, in our data set, transcripts for the taurine and polyamine transporters corresponded almost exclusively to *Gammaproteobacteria*, concretely to the Gammaproteobacteria_11 MAG. Interestingly, this organism seemed to express both machineries differently. While the *tauA* gene followed the “V” shape, *potF* had no transcription in winter, and its expression profile increased steeply in mid-June. The *potF* profile is likely associated with the phytoplankton bloom, since polyamines like putrescine and spermidine are by-products of phytoplankton decay, and their concentration can increase during bloom episodes (85, 86). Taurine, on the contrary, is also produced by metazoans and therefore might have higher concentrations in winter (84). If our transcriptomic data would reflect real taurine and polyamine utilization, *Pelagibacterales* might not always be the main marine organism taking up these substrates, and under certain conditions, some *Gammaproteobacteria* might play a major role. Interestingly, in Antarctic waters, a metaproteomic assessment found that most taurine transporters were affiliated to *Pelagibacterales,* suggesting that our results might not be circumscribed to a polar biogeography (51).

Similarly to taurine and polyamines, different studies have remarked on the predominance of *Alphaproteobacteria* (especially *Pelagibacterales* and *Rhodobacterales*) in the uptake of amino acids, which can serve as a nitrogen source in the ocean (51, 59, 88–90, 92). In our samples, *Alphaproteobacteria* also dominated the expression profiles of the amino acid uptake genes *livJ* and *bztA*, although the profiles showed a bigger diversity with a considerable fraction of the transcripts affiliating to *Gammaproteobacteria*, *Actinobacteriota,* and *Bacteroidota*. Both genes reached their highest expression in March, with *bztA* presenting higher levels of transcription. Different studies have shown that a considerable fraction of Arctic bacteria (10%–60%) can incorporate different amino acids into the cell during the whole year (93–96). Therefore, the high expression levels for *bztA* and *livJ* in March and June might indicate that amino acids are a widespread nutrient source in different periods, maybe when other nutrients are more limiting.

### An omics perspective of the nitrogen cycle in the Arctic

Our study tries to understand the cycling of nitrogen in an Arctic microbial community using metagenomic and metatranscriptomic data. Transcriptomics can provide invaluable information about microbial function, but it does not always reflect that a certain process is occurring. Therefore, the interpretation of our results is limited, especially due to the lack of rich environmental data. Thus, to support our results, we have searched for previous observations in marine data sets, especially those including Arctic samples.

Our study shows that nitrifying organisms, *Nitrosphaeria* and *Nitrospira*, actively expressed the corresponding machineries for ammonia and nitrite oxidation during winter, when they also transcribed genes for autotrophic growth. In fact, those nitrifying genes reached expression levels unmatched from any other nitrogen-cycling gene in our samples, with the exception of bacterial *amt* during the summer period. After winter, the expression of these genes plummeted, suggesting that nitrification is prevalent in the Arctic during winter and disappears in summer, as shown in different studies (14, 44, 45). In May, there is a change in the community composition associated with the phytoplankton bloom, also reflected in the expression profiles of many nitrogen-cycling genes, with a predominance of *Alphaproteobacteria, Gammaproteobacteria,* and *Bacteroidota* in the summer DNA and RNA libraries. Many of these organisms were probably thriving on the carbohydrates released during the phytoplankton bloom (23). For these bacteria, ammonia seemed to be the preferred nitrogen source based on the widespread presence and expression of the *amt* gene. However, many of the highly abundant organisms possessed several ways to incorporate nitrogen. We hypothesize that their ability to incorporate different nitrogen sources allowed them to thrive during the bloom in comparison to other bacteria. Previous studies have shown that heterotrophic bacteria are key players in the assimilation of different N sources during phytoplankton blooms (97) and that bacteria proliferating during blooms have higher proportions of proteins for uptake of organic nitrogen compared to other bacteria (98). Strikingly, for many genomes, we could only detect one mechanism or none (Fig. 5; Table S2). For instance, the MAG Alphaproteobacteria_10 affiliated to *Pelagibacterales* was the most abundant organism in all libraries, but did not encode any gene for nitrogen incorporation from the studied data set. This illustrates another limitation of our analysis since bioinformatic binning does not always recover complete genomes or can produce chimeric organisms. Therefore, some relevant genes might not have been binned and were missing from our analysis. As has been shown in other *Pelagibacterales* genomes (99), it is highly likely that the Alphaproteobacteria_10 MAG possessed at least one *amt* gene to incorporate ammonia that was not correctly binned. In fact, this MAG has a gene for a glutamine synthetase responsible for incorporating ammonia into the metabolism by forming glutamine (Table S6).

This diversity of transcribed strategies to assimilate nitrogen might suggest a strong competition for this nutrient during the phytoplankton bloom. We lacked biogeochemical data for most nitrogen species, but our measurement for nitrate and nitrite showed a decrease during the bloom period along with other nutrients (Fig. 1B). In fact, with the beginning of the bloom, genes like *nrtA*, *nasA*, or *potF* steeply increased their transcription levels from a winter situation with almost no expression. This might indicate that bloom microorganisms try to diversify their nitrogen sources, since some pools might be exhausted as previously suggested. For instance, a previous transcriptomic analysis during a phytoplankton bloom reported that bacteria express in cascade different mechanisms for utilization of organic matter, including nitrogen-rich compounds, influencing the availability of these substrates (100).

Interestingly, with the exception of ammonia transporters, which are widespread, different mechanisms seem to be present in specific groups and differentially expressed, suggesting a niche specialization. Thus, *Gammaproteobacteria* seem to monopolize organic nitrogen compounds like taurine and polyamines, *Bacteroidota* are specialized in assimilating nitrate, and *Alphaproteobacteria* genomes have higher expression of machineries to utilize urea and amino acids (Fig. 3). We hypothesize that specific bacterial groups have the ability to use different nitrogen sources during the summer bloom, which could indicate a taxonomic niche specialization for the utilization of specific nitrogen species. This kind of niche differentiation for major phylogenetic groups has been suggested for the uptake of low molecular weight organics (101).

Our study highlights the dynamics of the nitrogen cycle in the Canadian Arctic using omics approaches. There was a succession from a winter scenario with nitrifying communities actively transcribing their machineries to a summer situation dominated by bloom-dependent bacteria, which expressed different mechanisms to assimilate nitrogen. In fact, summer bacteria seem to specialize in different nitrogen compounds according to gene expression, suggesting a niche differentiation for the most abundant organisms to avoid competition. Further studies incorporating biogeochemical data are needed to validate this hypothesis and fully understand the cycling of nitrogen in the Arctic.

## MATERIALS AND METHODS

### Sampling, nucleic acid extraction, and sequencing

The sampling campaign, nucleic acid extraction, and sequencing were already described in a previous publication from Puente et al. (22). In short, samples were collected in Dease Strait, lower Northwest Passage, Nunavut, Canada (69.03°N, 105.33°W; Fig. S1), from March until July 2014 within the 2014 Ice Covered Ecosystem-CAMbridge Bay Process Study. Most water samples were collected below first-year ice (1.8 m–2.1 m thick) at a depth of 2.5 m. The samples for DNA and RNA were successively filtered with 20, 3, and 0.22 µm filters. Here, we present results from the 0.22 µm filters. A list of the different samples for DNA and RNA libraries can be found in Table S1. DNA was extracted using a modified version of the phenol/chloroform protocol (102), while RNA was obtained using Qiagen’s RNeasy kit without rRNA depletion. Sequencing was performed for most of the samples at CNAG on an Illumina HiSeq 2000 sequencing platform using a TruSeq paired-end cluster kit, v.3. Environmental data (including chlorophyll *a*, nitrate, nitrite, phosphate, and silicate, Tables S3 and S4) were sampled as described in Campbell et al. (103).

### Assembly, binning, and metabolic prediction

Read correction of the corresponding libraries was performed with Trimmomatic using standard parameters (104). We used SPAdes v.3.15.5 (105) to perform a *de novo* individual assembly for each of the metagenomics libraries. Afterward, we performed binning using a combination of Metabat2 v.2.15 (106) and Maxbin v.2.2.7 (107) with final processing with DasTool v.1.1.2 (108). We used GTDB-tk v.2.1.1 (109) and checkM v.1.1.3 (110) to evaluate the quality and taxonomy of the obtained bins. We combined all the bins from the different individual assemblies and dereplicated them using the dereplication feature of CoverM v.0.6.1 (https://github.com/wwood/CoverM) with default settings except the flags “--checkm-tab-table --dereplication-ani 95 --dereplication-prethreshold-ani 90.” Dereplicated bins were further refined via a targeted reassembly pipeline as described in Laso-Pérez et al. (111). In short, metagenomic reads were mapped using bbmap (https://sourceforge.net/projects/bbmap/) to the bins. These reads were used for a new assembly with SPAdes v.3.15.5 and filtering contigs below 1,500 bp. This procedure was iterated for several rounds for each bin until no further improvement was obtained. We assessed the bin quality using checkM and checkM2 v.1.0.1 (112) by considering completeness, contamination, N50 value, and number of scaffolds. Only bins with 50% completeness and contamination below 10% were selected for further analysis and considered as MAGs. To calculate the MAG relative abundance in our metagenomics and metatranscriptomics samples, we used CoverM v.0.6.1 with all the final MAGs. We also recruited the 16S rRNA gene reads from the DNA and RNA libraries to then classify them based on SSU SILVA release 138 (113) using phyloFlash v.3.4.2 (114). Results were also visualized in R v.4.2.1 (https://www.R-project.org/). We used the SqueezeMeta pipeline v.1.5.1 (115) for automatic metabolic prediction of the MAGs and calculation of coverage and abundance estimations of the predicted genes in the different data sets, including calculations of transcripts per million reads (TPM) by competitive mapping of the reads. Multivariate statistics of the transcriptomic data were performed in R v.4.2.1 using the package vegan (116). The corresponding script can be found at https://github.com/gecko1990/Arctic_N_cycle.

### Identification of nitrogen-cycling genes and classification

To study the cycling of nitrogen, we developed a database of phylogenetic trees for different marker genes according to the pipeline described in Rivas-Santisteban et al. (117) and available in a GitHub repository (https://github.com/Robaina/MetaTag). In this pipeline, high-quality trees are constructed to distinguish between true marker genes and closely related paralogs with different functions. Then, candidate genes from the different query MAGs are retrieved using specific protein models and databases (i.e., COGs, arCOGs, and KEGG), and they are classified within the previously constructed high-quality tree, allowing their classification as true marker genes or a closely related paralog. We built reference databases for 18 genes encoding different proteins involved in the nitrogen cycle (Table S7) including *amoA, nifH, nirK, ureC, uca, narG, nxrA, nirS, nasA, nosZ, hzsA, amt, nrtA, tauA, potF, livJ, bztA,* and *ilvC*. After tree construction, we searched for the candidate genes in all the MAGs based on the predictions of SqueezeMeta according to specific protein model annotations (Table S7) to then place those query sequences into the phylogenetic trees and distinguish between the true marker genes and closely related paralogs, keeping the true marker genes. Additionally, we searched for *urtA*, *ureA, ureB, amoB,* and *amoC* genes using specific protein model annotations (Table S7), but without performing phylogenetic placement. For these genes, we extracted their coverage and abundance estimations calculated by the SqueezeMeta pipeline to visualize the temporal and taxonomic changes in our samples by using the R v.4.2.1 software. We calculated the RNA:DNA ratio by dividing the corresponding RNA and DNA values for each gene. For the RNA samples of March 11 and May 15, we used the average DNA TPM values from the 9th and 13th of March and the 10th and 19th of May, respectively. Gene queries can be found in the corresponding text files: Data S1 (nitrogen-cycling genes), Data S2 (*nifH*-like gene and corresponding operon from Alphaproteobacteria_22 MAG and *glnA* gene from Alphaproteobacteria_10 MAG), and Data S3 (*nifH*-like genes in the unbinned fraction).

### Identification of autotrophic genes and classification

To study the presence of autotrophic genes, we used the HMM models for marker genes of carbon fixation pathways developed by Garritano et al. (118) and deposited in their GitHub repository (https://github.com/alegarritano/HMM_CFP/blob/main/CFP.HMM). We searched for the corresponding gene using HMMER (http://hmmer.org/) with the command “hmmsearch -E 1e-99.” Furthermore, we searched for additional genes related to carbon fixation, looking for specific protein motifs in the annotations provided by SqueezeMeta (Table S7). Gene queries can be found in Data S4.

## Data Availability

Generated sequences were deposited under NCBI BioProject ID PRJNA803814. The resulting MAGs were deposited under NCBI BioProject ID PRJNA1044758.

## SUPPLEMENTAL MATERIAL

Data S1 mbio.00749-25-s0001.txt Query nitrogen-cycling genes.

Data S2 mbio.00749-25-s0002.txt *nifH*-like genes Alphaproteobacteria_22 MAG and *glnA* gene Alphaproteobacteria_10 MAG.

Data S3 mbio.00749-25-s0003.txt *nifH*-like genes from the unbinned fraction.

Data S4 mbio.00749-25-s0004.txt Query autotrophic genes.

Figure S1 mbio.00749-25-s0005.pdf Sampling location.

Figure S2 mbio.00749-25-s0006.pdf Phyloflash analysis.

Figure S3 mbio.00749-25-s0007.pdf RNA/DNA ratio.

Figure S4 mbio.00749-25-s0008.pdf Scarce gene TPM abundances.

Figure S5 mbio.00749-25-s0009.pdf Autotrophy genes.

Supplemental Tables mbio.00749-25-s0010.xlsx Tables S1-S8.

ASM does not own the copyrights to Supplemental Material that may be linked to, or accessed through, an article. The authors have granted ASM a non-exclusive, world-wide license to publish the Supplemental Material files. Please contact the corresponding author directly for reuse.
