# Supplementary figures and images for: Nitrogen cycling during an Arctic bloom: from chemolithotrophy to nitrogen assimilation
Source: mBio. 2025 May 12;16(6):e00749-25. doi: 10.1128/mbio.00749-25 (PMC12153308; doi:10.1128/mbio.00749-25)

**Figure S1. Map of the sampling location at the Dease Strait.**

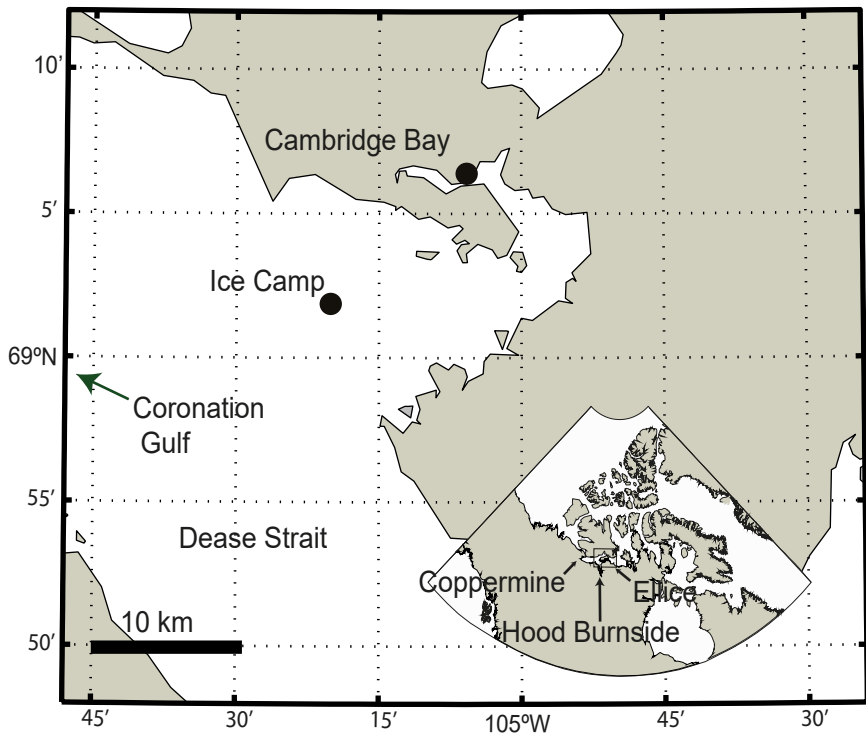

Supplement: Figure S1 — Sampling location. [file mbio.00749-25-s0005.pdf]
