# Supplementary material for: Nitrogen cycling during an Arctic bloom: from chemolithotrophy to nitrogen assimilation
Source: mBio. 2025 May 12;16(6):e00749-25. doi: 10.1128/mbio.00749-25 (PMC12153308; doi:10.1128/mbio.00749-25)

Figure S2. Taxonomic affiliation of the 16S rRNA gene reads in the DNA (A) and RNA (B) libraries. The category "Other" represents clades recruiting less than 0.5% of the total reads. The data for these plots was obtained using phyloflash.

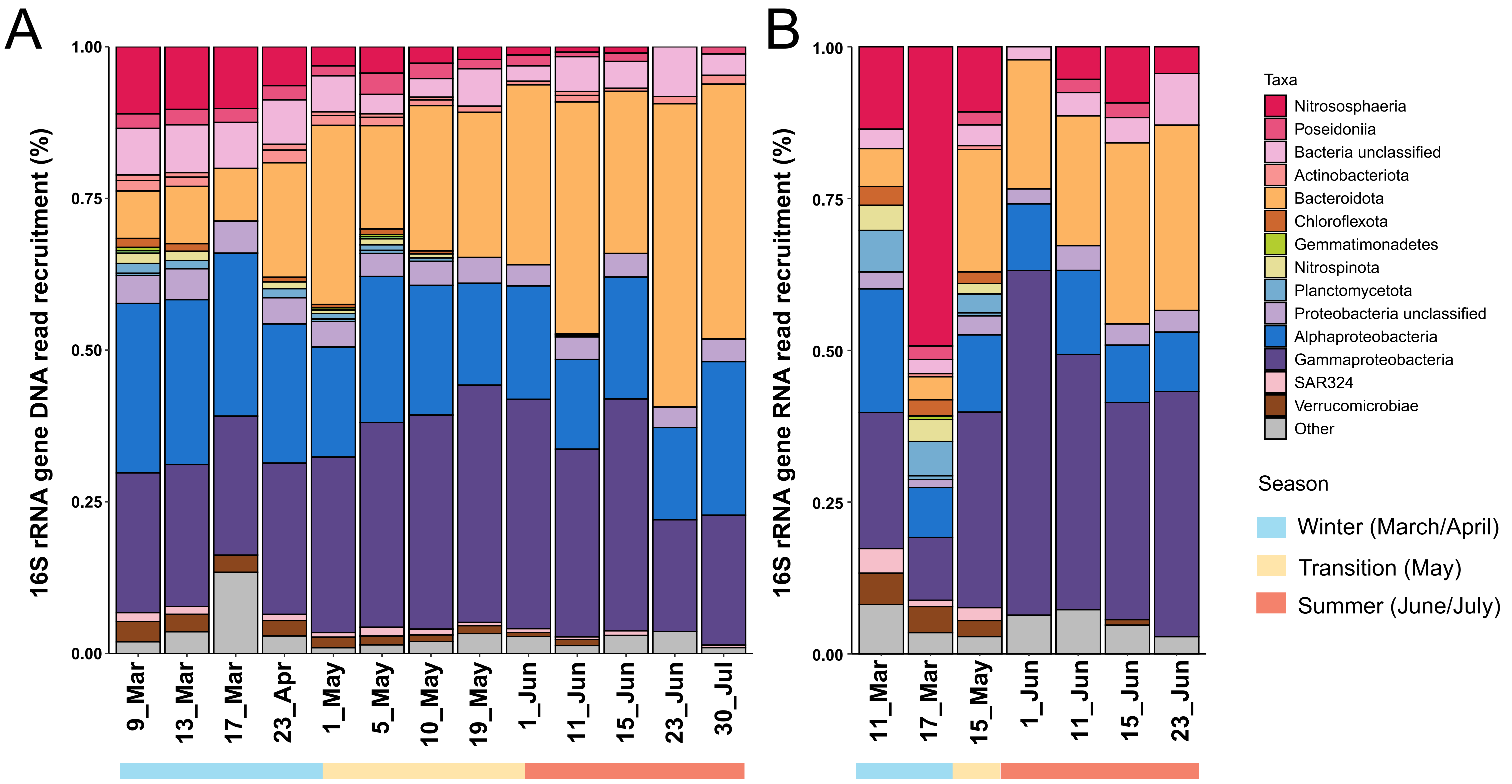

Supplement: Figure S2 — Phyloflash analysis. [file mbio.00749-25-s0006.pdf]
