# Supplementary material for: Nitrogen cycling during an Arctic bloom: from chemolithotrophy to nitrogen assimilation
Source: mBio. 2025 May 12;16(6):e00749-25. doi: 10.1128/mbio.00749-25 (PMC12153308; doi:10.1128/mbio.00749-25)

Figure S3. RNA/DNA ratio of the TPM values of nitrogen-cycling genes. Ratios are grouped at the class/phylum taxonomical levels.

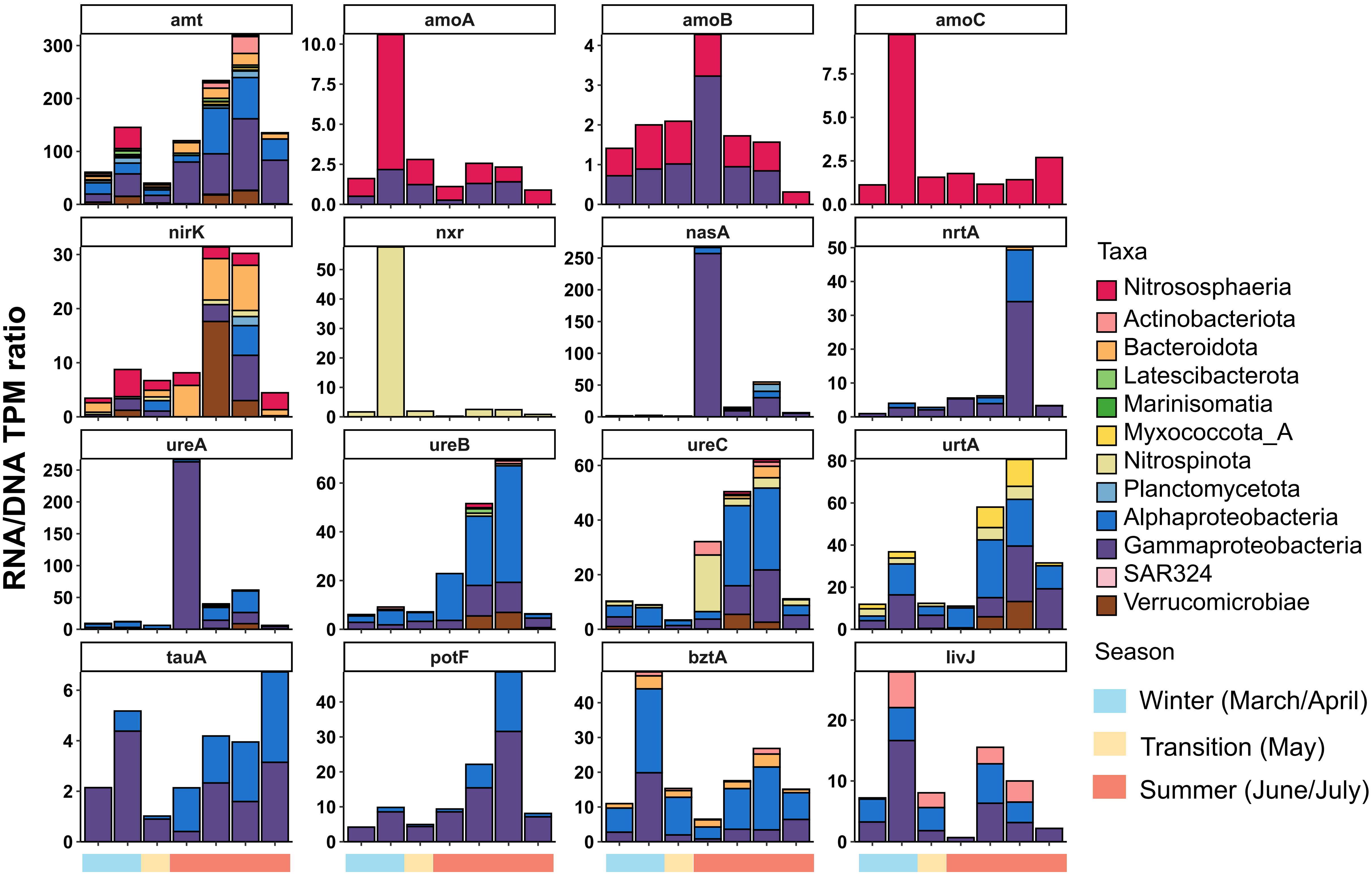

Supplement: Figure S3 — RNA/DNA ratio. [file mbio.00749-25-s0007.pdf]
