# Supplementary material for: Nitrogen cycling during an Arctic bloom: from chemolithotrophy to nitrogen assimilation
Source: mBio. 2025 May 12;16(6):e00749-25. doi: 10.1128/mbio.00749-25 (PMC12153308; doi:10.1128/mbio.00749-25)

Figure S4. TPM values of nitrogen-cycling genes with low values and the biosynthetic ilvC gene. A Total TPM in the DNA libraries of the different genes grouped by taxonomical class or phylum. **B** Total TPM in the RNA libraries of the different genes grouped by taxonomical class or phylum.

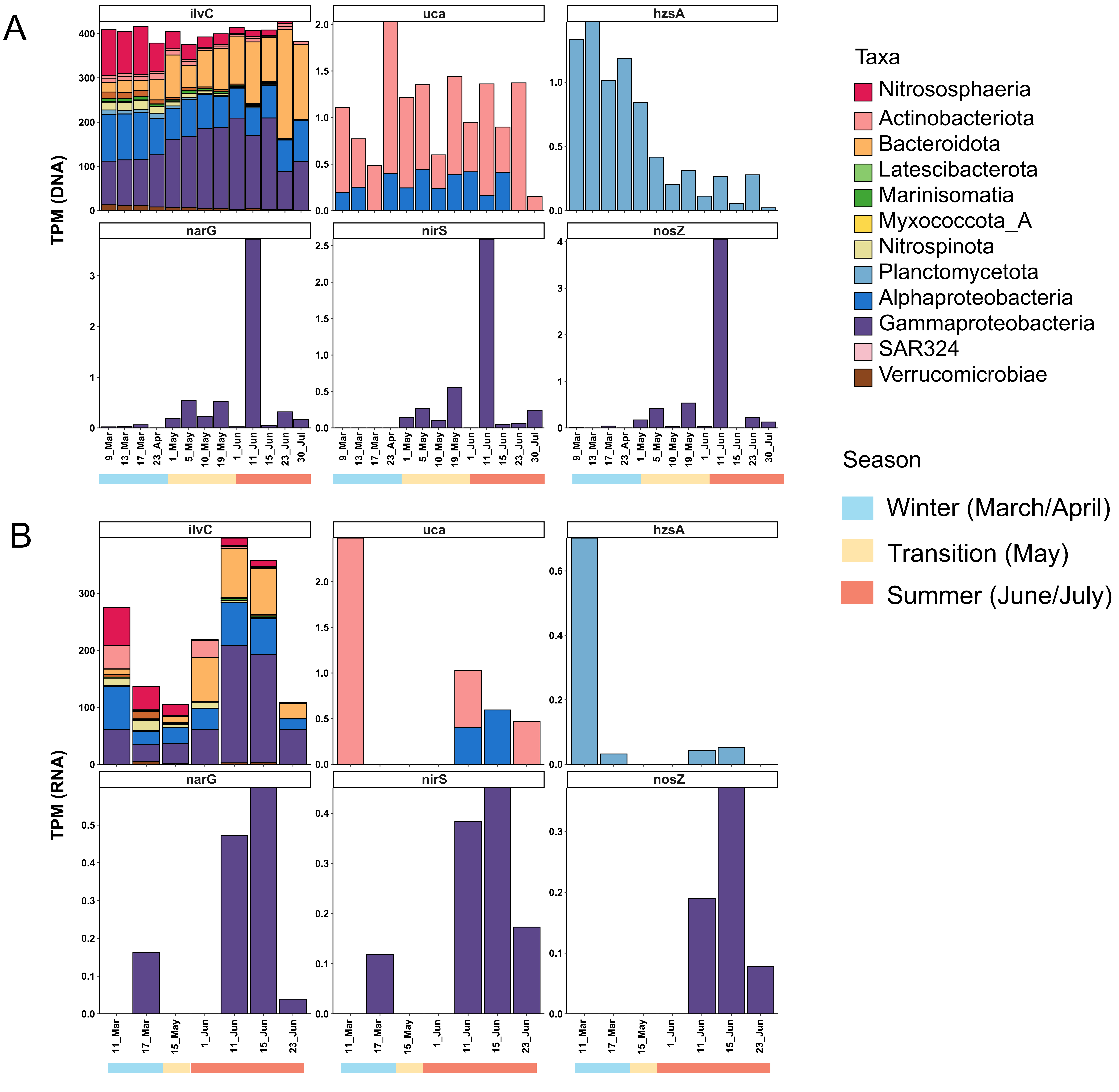

Supplement: Figure S4 — Scarce gene TPM abundances. [file mbio.00749-25-s0008.pdf]
