# Supplementary material for: Nitrogen cycling during an Arctic bloom: from chemolithotrophy to nitrogen assimilation
Source: mBio. 2025 May 12;16(6):e00749-25. doi: 10.1128/mbio.00749-25 (PMC12153308; doi:10.1128/mbio.00749-25)

Figure S5. Expression of autotrophic genes based on TPM values. Genes are grouped at class or phylum level. CBB: Calvin–Benson–Bassham cycle; rbcL/S: rubisco large and small subunit; PKK: phosphoribulokinase; rTCA: reverse tricarboxylic acid cycle; HBD: 4-hydroxybutyrate dehydrogenase from the 4-hydroxybutyrate/3-hydroxypropionate (4HB/3HP) cycle; TIGR04253: motif from the mesaconyl-CoA isomerase (mct) of 3-hydroxypropionate (3HP) bi-cycle and for the (hbd) from 4 -hydroxybutyrate/3-hydroxypropionate (4HB/3HP) cycle.

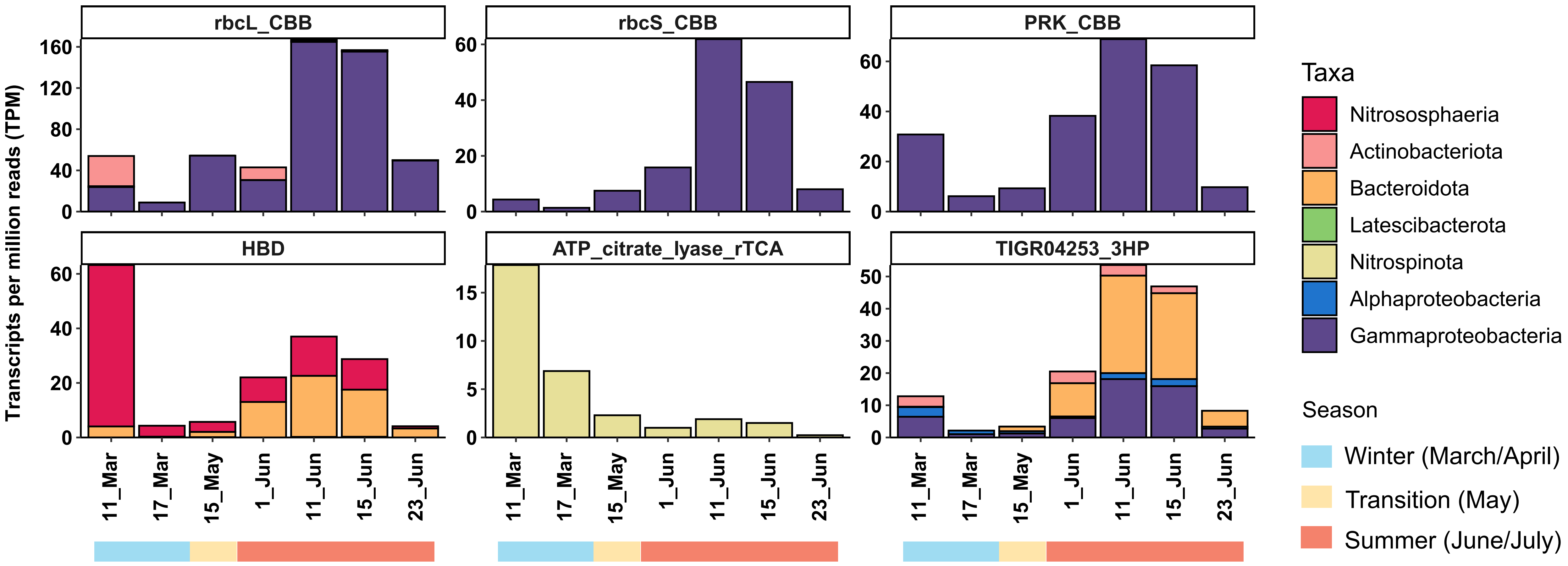

Supplement: Figure S5 — Autotrophy genes. [file mbio.00749-25-s0009.pdf]
